# PPS, a Large Multidomain Protein, Functions with Sex-Lethal to Regulate Alternative Splicing in *Drosophila*

**DOI:** 10.1371/journal.pgen.1000872

**Published:** 2010-03-05

**Authors:** Matthew L. Johnson, Alexis A. Nagengast, Helen K. Salz

**Affiliations:** Department of Genetics, Case Western Reserve University, School of Medicine, Cleveland, Ohio, United States of America; University of Illinois at Urbana-Champaign, United States of America

## Abstract

Alternative splicing controls the expression of many genes, including the *Drosophila* sex determination gene *Sex-lethal* (*Sxl*). *Sxl* expression is controlled via a negative regulatory mechanism where inclusion of the translation-terminating male exon is blocked in females. Previous studies have shown that the mechanism leading to exon skipping is autoregulatory and requires the SXL protein to antagonize exon inclusion by interacting with core spliceosomal proteins, including the U1 snRNP protein Sans-fille (SNF). In studies begun by screening for proteins that interact with SNF, we identified PPS, a previously uncharacterized protein, as a novel component of the machinery required for *Sxl* male exon skipping. PPS encodes a large protein with four signature motifs, PHD, BRK, TFS2M, and SPOC, typically found in proteins involved in transcription. We demonstrate that PPS has a direct role in *Sxl* male exon skipping by showing first that loss of function mutations have phenotypes indicative of *Sxl* misregulation and second that the PPS protein forms a complex with SXL and the unspliced *Sxl* RNA. In addition, we mapped the recruitment of PPS, SXL, and SNF along the *Sxl* gene using chromatin immunoprecipitation (ChIP), which revealed that, like many other splicing factors, these proteins bind their RNA targets while in close proximity to the DNA. Interestingly, while SNF and SXL are specifically recruited to their predicted binding sites, PPS has a distinct pattern of accumulation along the *Sxl* gene, associating with a region that includes, but is not limited to, the *SxlPm* promoter. Together, these data indicate that PPS is different from other splicing factors involved in male-exon skipping and suggest, for the first time, a functional link between transcription and SXL–mediated alternative splicing. Loss of zygotic PPS function, however, is lethal to both sexes, indicating that its role may be of broad significance.

## Author Summary

In *Drosophila* the sex-specific ON/OFF regulation of *Sex-lethal (Sxl)* is controlled by an autoregulatory splicing mechanism that depends on the SXL protein interacting with general splicing factors. Here we identify PPS as a novel component of the machinery required for *Sxl* splicing autoregulation by showing that the lack of *pps* function interferes with *Sxl* expression and that the PPS protein is physically linked to the *Sxl* pre–mRNA, the SXL protein and components of the general splicing machinery. PPS, however, stands apart from all other proteins known to control *Sxl* splicing because it is not a general splicing factor. Furthermore, PPS has a distinct pattern of accumulation along the *Sxl* transcription unit that suggests PPS is loaded onto the RNA at the promoter. Together with the observation that the PPS protein contains four signature motifs typically found in proteins that function in transcriptional regulation, our data suggest that linking transcription to splicing regulation is important for controlling *Sxl* expression. This idea is especially intriguing because it indicates that the coupling of transcription and splicing seen *in vitro* and in cell culture studies is likely to be pertinent to developmentally controlled patterns of gene expression in the living animal.

## Introduction

Understanding tissue- and stage-specific gene regulation remains one of the central issues in developmental biology. Studies of developmentally important genes, such as those that specify and maintain cell fate, have revealed that many genes are regulated post-transcriptionally. The *Drosophila* sex-determination gene *Sex-lethal (Sxl)* is a prime example of a developmental switch gene regulated by alternative splicing. Throughout most of development and in adult tissues, *Sxl* is controlled by sex-specific alternative splicing to produce mRNAs with different coding potentials [1]. In males, all transcripts include the translation-terminating third exon leading to the production of mRNAs that encode truncated, inactive proteins. In females, the third exon is always skipped to generate protein encoding mRNAs. The mechanism leading to exon skipping is autoregulatory and depends on the SXL protein binding to multiple intronic sites located both upstream and downstream of the regulated exon. Current models, based on both biochemical and genetic studies, suggest that SXL forces the male exon to be skipped by interacting with and antagonizing a set of general splicing factors, including the U1 snRNP, the U2AF heterodimer, FL(2)d and SPF45 [2]–[4]. Because *Sxl* controls both its own expression and the expression of a set of downstream target genes, this autoregulatory splicing loop serves as a heritable and irreversible molecular switch for the developmental pathways controlling both somatic sex determination and X-chromosome dosage compensation.

Initiation and stable engagement of the *Sxl* autoregulatory splicing loop requires the coordinated use of two alternative promoters [5]–[7]. Throughout most of development, *Sxl* is expressed from the non-sex specific “maintenance” promoter, *SxlPm*. *SxlPm* is first expressed during the maternal to zygotic transition, but prior to that time *Sxl* is transiently expressed from the female-specific “establishment” promoter, *SxlPe*. The *SxlPe*-derived transcripts, unlike the transcripts produced from *SxlPm*, are spliced by default to produce SXL protein. Thus the SXL protein present in XX embryos when *SxlPm* is first activated serves to drive the initiating round of exon skipping which leads to a self-sustaining splicing loop. In XY animals, on the other hand, *SxlPe* is not activated, there is no SXL protein, and all *SxlPm-*derived transcripts are spliced in the male mode. While coordinated promoter switching is critical for successful establishment of the *Sxl* autoregulatory splicing loop in early embryogenesis, it has been generally assumed that transcription plays little, if any, role in sex-specific regulation after this point.

Here we report the identification and analysis of a previously uncharacterized protein, named Protein Partner of Sans-fille (PPS, CG6525), as a novel component of the machinery that controls *Sxl* alternative splicing. PPS, a large multidomain protein classified as a transcription regulator based on the presence of 4 distinct and conserved sequence motifs, was identified in a yeast two hybrid screen for proteins that interact with Sans-fille (SNF), the *Drosophila* homolog of the U1 snRNP protein, U1A. We provide compelling evidence that PPS has a direct role in *Sxl* male exon skipping by showing first that the loss of *pps* function interferes with *Sxl* function, and second that PPS can form a complex with the U1 snRNP, SXL and the *Sxl* pre-mRNA. In addition, we mapped the association of PPS, SXL and SNF along the *Sxl* gene by chromatin immunoprecipitation (ChIP), providing evidence that these proteins, like many other splicing factors, bind their RNA targets while in close proximity to the DNA. While we found that SXL and SNF associate with their predicted binding sites, PPS has a distinct pattern of accumulation along the *Sxl* gene which suggests that PPS is loaded onto the RNA at the promoter. Finally, we show that PPS function is not restricted to *Sxl* splicing regulation, indicating that PPS is likely to be more broadly involved in development.

## Results

### Identification of PPS, a SNF–interacting protein

**Figure 1:**
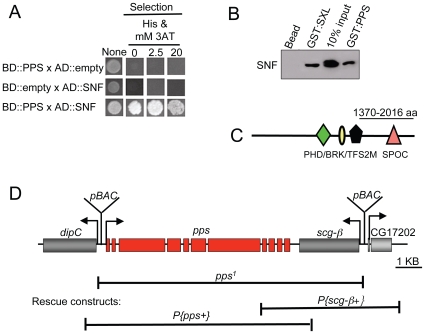
PPS, a large multidomain protein, is a SNF interacting protein. (A) Yeast two-hybrid interactions between PPS and SNF. Positive interactions were tested by assaying the ability of the transformed yeast to grow on selective media after 3 days. (B) PPS/SNF complex assembly tested by GST pull-down assays in whole cell extracts. The lane marked 10% input is a control in which the amount of extract corresponds to 10% of the material applied to the glutathione affinity beads. (C) Diagram of the 2016 amino acid PPS protein. PPS contains 4 conserved motifs, which are drawn approximately to scale. The line above the diagram is the region of the protein used for the yeast two hybrid experiments in (A), in the GST pulls experiments in (B) and for production of the PPS antibody. (D) Genomic organization of PPS and its neighboring genes. Solid boxes represent exons. The position of the insertions used to generate *pps^1^* is indicated above the diagram. The genomic DNA deleted in *pps^1^* and the genomic DNA used in the rescue constructs is indicated by a solid line below the diagram.

CG6525 was identified in a yeast two hybrid screen for SNF-interacting proteins, giving the gene its name ***p*** *rotein* ***p*** *artner of* ***s*** *ans-fille (pps;* Figure 1A). To demonstrate that the PPS/SNF interaction also occurs in *Drosophila* cell extracts, we assayed for complex formation by pull-down experiments in which a GST fusion protein containing the C-terminal end of PPS (amino acids 1370–2016) was expressed in *E. coli*, bound to glutathione sepharose beads, and incubated with protein extracts made from embryos. The presence or absence of SNF in the complex formed on the beads was assayed by Western blot analysis (Figure 1B). In control studies, we used a GST::SXL fusion protein since it is known to form a complex with SNF [2]. As predicted by the two hybrid data, we found that GST::PPS, but not GST alone, was capable of selecting SNF out of extracts as efficiently as GST::SXL. These data therefore confirm that PPS and SNF associate *in vivo*.

PPS is located on the 3rd chromosome (87B) and, in agreement with the predicted gene structure, we found that the *pps* transcription unit extends over 6.7 kb. and the 11 constitutively spliced exons are predicted to encode an uncharacterized 2016 amino acid protein (Figure 1C and 1D). The *pps* open reading frame contains 4 conserved motifs: PHD finger (plant homeodomain), BRK (Brahama and Kismet), TFS2M (transcription elongation factor S-II middle) and SPOC (Spen paralogue and orthologue C-terminal). According to the Gene Ontology Database, which assigns functions to uncharacterized proteins based the presence of sequence motifs, PPS is likely to function in transcriptional regulation (see discussion).

### *pps* is an essential gene

To gain insight into the biological role of PPS, we generated a molecular null allele using an FRT-based targeted deletion strategy [8],[9]. Briefly, we induced recombination in animals heterozygous for two FRT-bearing piggyBac insertions with controlled expression of the FLP recombinase and identified a deletion with the desired endpoints using a PCR based strategy. The resulting deletion, depicted in Figure 1D, removes the entire coding sequence of *pps* as well as the adjacent gene, *Scg-β*. Animals homozygous for this two gene deletion die during the third instar larval stage. Two critical experiments demonstrate that the lethality is due to the loss of *pps* and not *Scg-β*. First, lethality was fully rescued by one or two copies of *P{pps+}*, a genomic transgene that carries just the *pps* gene (90%, n = 554). Second, all aspects of the mutant phenotype remained unchanged by the addition of multiple copies of the adjacent *P{Scg-β+}* genomic transgene (see Materials and Methods for details). Thus, these data provide strong evidence that disruption of PPS is responsible for the larval lethal phenotype and the two gene deletion we have isolated behaves as a *pps* null allele. Based on these genetic data, we have named this deletion *pps^1^*.

Homozygous *pps^1^* mutant animals fail to survive to adulthood, although all animals reach the third instar larval stage. Consistent with the failure to pupate, mutant third instar larvae were found to have a number of defects, including small, underdeveloped imaginal discs, abnormal polytene chromosome morphology and melanized patches of tissue that resemble melanotic tumors (data not shown).

Although *pps* null mutants complete embryogenesis without any apparent defects, we cannot rule out an earlier function in embryogenesis. PPS is a maternally provided protein and the extended stability common to many maternally provided proteins typically result in the rescue of homozygous mutant animals into the larval stages. Thus, *pps* mutant animals may survive until the maternal stores of protein are depleted, masking a potential requirement in embryogenesis.

### Incomplete rescue of *pps^1^* reveals a role in *Sxl* regulation

**Figure 2:**
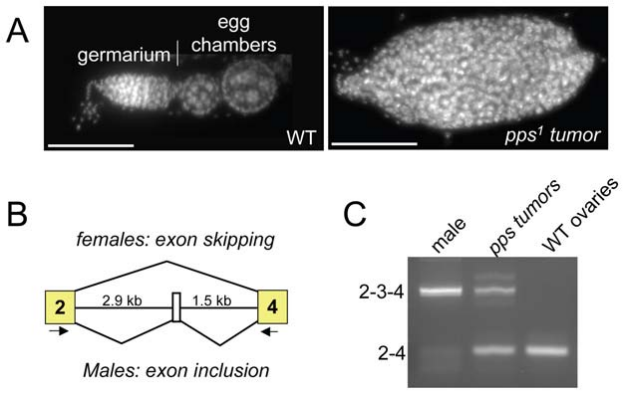
*Sxl* splicing is disrupted in the ovaries of incompletely rescued *pps^1^* mutant females. (A) DAPI-stained ovariole from a wild female (WT) and a *P{pps+}/+*; *pps^1^/Df(3R)Exel7316* female. (B) Diagram of the alternative splicing event that produces sex-specific *Sxl* transcripts. The arrows below the diagram indicate the position of the PCR primer pairs used for RT–PCR. (C) The tumor phenotype is correlated with *Sxl* splicing defects. Splicing was assayed by RT–PCR using RNA isolated from ovaries dissected from *P{pps+}/+*; *pps^1^/Df(3R)Exel7316* females (*pps* tumors). Controls include splicing in ovaries isolated from wild type (WT) females and splicing in adult males.

During the course of this analysis, we noted that, while either one or two copies of the *P{pps+}* transgene was sufficient to rescue the lethality of *pps^1^* homozygous mutant females, two copies were necessary to rescue the females to fertility. An examination of the ovaries isolated from these sterile mutant females revealed that the ovaries contained tumors (Figure 2A). Ovarian tumor phenotypes are also observed in partial loss of function *snf* mutant backgrounds, where the phenotype is caused by defects in *Sxl* splicing regulation [2],[10]. To investigate the possibility that the *pps* tumor phenotype is also correlated with *Sxl* misregulation, we used RT-PCR to assay the *Sxl* RNA products present in isolated ovarian tissue. Using a single primer pair capable of detecting the female and the larger male spliced products, we found that in ovarian tissue isolated from sterile mutant females, a significant proportion of the spliced products contained the male-specific exon (Figure 2B and 2C). Thus, based on these partial loss of function mutant phenotypes, we conclude that *pps*, like *snf*, is required to achieve stable *Sxl* activity in the female germline.

### *pps^1^* is a maternal effect modifier of *Sxl*

**Figure 3:**
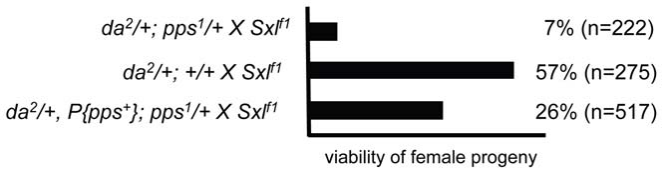
*pps* is a dosage-sensitive maternal modifier of *Sxl*. Synergistic genetic interactions lead to female lethality. In these assays, females of the indicated genotype were mated to *Sxl^f1^/Y* and the resulting progeny scored. On the assumption that an equal number of male and female progeny will be generated from each cross, the percent female viability was calculated by comparing the number of females recovered with the number of males recovered.

Activation of *Sxl* in the embryo is a multi-step process, starting with the coordinated use of two promoters and culminating with successful engagement of the autoregulatory splicing loop. Thus, perturbation of any single step in the process can lead to a defect in alternative splicing. As a consequence, embryos heterozygous for the normally recessive null allele of *Sxl (Sxl^f1^/+)* are particularly sensitive to the supply of specific splicing and transcription factors deposited into the egg by the mother (*e.g.* [2]–[4]). We therefore reasoned that if maternally provided PPS protein is important for any aspect of *Sxl* regulation, we might expect the viability of *Sxl^f1^/+* females to be affected if their mothers were heterozygous for *pps* (*pps^1^/+*). However, we found that these *Sxl^f1^/+* females were as viable as their control siblings (data not shown). To increase the sensitivity of this assay, we introduced a mutant allele of *daughterless (da^2^)* into the genetic background. *da* encodes a maternally supplied transcription factor required to activate *Sxl* [11],[12]. We chose *da^2^* to sensitize the genetic background because we have previously shown that the genetic interaction between *snf* and *da* is particularly strong [13]. In control crosses, we found that 57% of the expected *Sxl^f1^/+* daughters from *da^2^/+* mothers survived to adulthood (n = 275; Figure 3). However, when the mothers were heterozygous for both *pps^1^* and *da^2^*, there was a significant reduction in viability with only 7% of the expected *Sxl^f1^/+* daughters surviving to adulthood (n = 222). Restoration of female viability by the genomic rescue construct *P{pps+}* indicates that this female-lethal synergistic interaction is due to the loss of *pps* function (26%; n = 517).

To confirm the genetic relationship between *pps* and *Sxl*, we looked for synergistic interactions with mutant alleles of *fl(2)d*, *U2af38* and *spf45*. Mutations in these three genes were picked because they encode core spliceosomal proteins known to play an important role in *Sxl* autoregulation [2]–[4]. These data show that *pps^1^* in combination with mutations in each of these spliceosomal genes exerts a detrimental synergistic effect on the viability of *Sxl^f1^/+* females (Table 1). Together, these data indicate that the maternally provided PPS protein contributes, in some way, to *Sxl* regulation.

**Table 1:** Lethal interactions between *Sxl^f1^, pps^1^*, and mutations in core spliceosomal proteins.

Maternal Genotype	Viability of *Sxl^f1^/*+ female progeny
*fl(2)d^2^/+; pps^1^/+*	29% (n = 140)
*U2af38^ΔE18^/+; pps^1^/+*	12% (n = 154)
*spf45^Δ^/+; pps^1^/+*	18% (n = 140)

Females of the indicated maternal genotype were mated to *Sxl^f1^* males and the resulting progeny scored. The viability of the female progeny, all of which were heterozygous for *Sxl* (*Sxl^f1^/+*), was assessed by comparing the number of females recovered to the number of males recovered (n). Female progeny from control crosses of single mutant heterozygous mothers mated to *Sxl^f1^* males were as viable as their control siblings (data not shown).

### PPS associates with the U1 snRNP and the SXL protein

**Figure 4:**
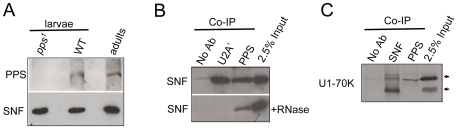
PPS associates with SNF and U1-70K in embryonic extracts. (A) *pps^1^* is a protein-null allele. Western blot of extracts made from wild type and *pps^1^* mutant animals probed with antibodies against PPS. SNF is used here as a loading control. (B) PPS interacts with SNF in a RNA–independent manner. Western blots of PPS and U2A' immunoprecipitations (Co-IP) in nuclear extracts made from embryos probed with an antibody against SNF. The RNase sensitivity of this association was tested by pretreating the extract with a combination of RNase A and RNase T1. Controls include the previously described RNase sensitive SNF/U2A' association. (C) PPS associates with U1-70K. Western blot of PPS and SNF immunoprecipitations (Co-IP) in nuclear extracts made from embryos probed with an antibody against U1-70K. Controls include the previously described SNF/U1-70K association. The lanes marked 2.5% input are controls in which the amount of extract corresponds to 2.5% of the material used in each Co-IP experiment.

Previous studies have shown that SXL interacts with SNF in the context of the U1 snRNP [2]. We reasoned, therefore, that if *pps* has a direct role in *Sxl* splicing autoregulation, then we might be able to detect physical interactions between PPS, the U1 snRNP and SXL. To test this, we generated an antibody against the C-terminal end of PPS (amino acids 1370–2016) for co-immunoprecipitation assays. PPS is predicted to encode a single polypeptide of 222 kD, and as predicted, we found that on Western blots, the wild type protein migrates at about 220 kD in extracts made from adults of both sexes, embryos and third instar larvae (Figure 4A, and data not shown). In contrast, no immunoreactivity was detected in extracts made from third instar larvae homozygous for *pps^1^*, demonstrating the specificity of this antibody. Using this antibody for co-immunoprecipitation experiments, we were able to confirm that PPS and SNF associate *in vivo* (Figure 4B). As expected, RNase addition did not abrogate the SNF/PPS interaction, even though the RNase treatment was sufficient to disrupt the known RNase-sensitive interaction between SNF and U2A' (Figure 4B).

To test whether PPS associates with SNF as a component of the U1 snRNP, we asked whether we could detect an interaction between PPS and another core U1 snRNP protein, U1-70K. Our data shows that we were able to co-immunoprecipitate PPS and U1-70K (Figure 4C), although we noted that PPS seems to preferentially associate with the more slowly migrating U1-70K species, among the major U1-70K isoforms observed in whole cell extracts. U1-70K is a phosphorylated protein, and studies in mammalian cells that have shown that dephosphorylation of U1-70K is necessary for the splicing reaction to proceed [14]. Thus, if PPS does in fact preferentially associate with the highly phosphorylated form of U1-70K, our data would lead to the conclusion that PPS, unlike SNF, only transiently associates with the U1 snRNP. Direct support for this conclusion comes from our more detailed analysis of PPS's role in *Sxl* splicing autoregulation described below.

**Figure 5:**
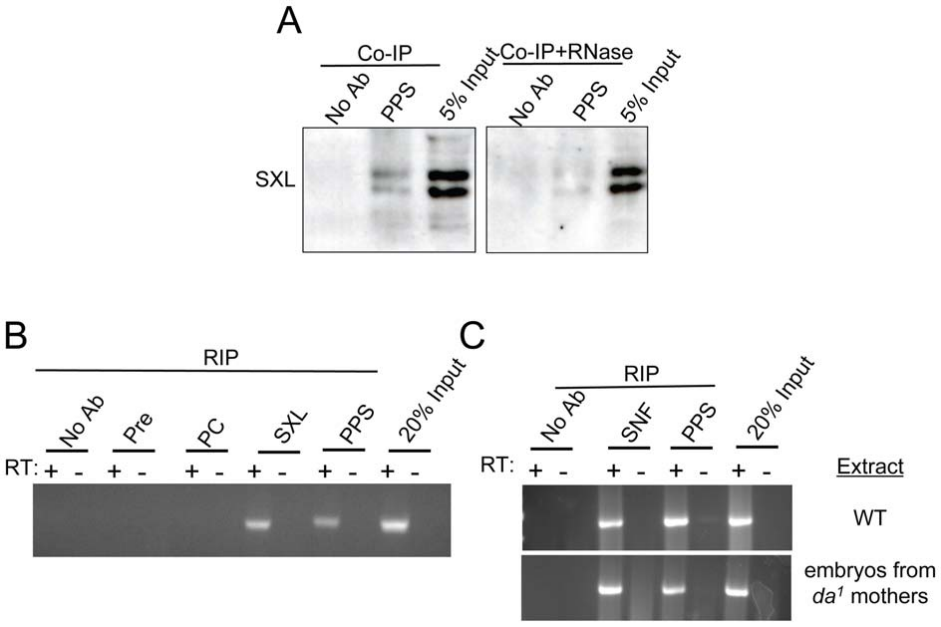
PPS associates with the SXL protein and the *Sxl* pre–mRNA. (A) PPS interacts with SXL in a RNA-dependent manner. Western blots of PPS immunoprecipitations (Co-IP) in nuclear extracts made from embryos probed with an antibody against SXL. The RNase sensitivity of this association was tested by pretreating the extract with a combination of RNase A and RNase T1. (B,C) PPS associates with the unspliced *Sxl* pre-mRNA in a SXL-independent manner. RNA-immunoprecipitation assays (RIP) were carried out in nuclear extracts made from wild type embryos (WT), or embryos from *da^1^/da^1^* mothers. The presence of unspliced *Sxl* RNA in the IP pellet was detected by RT-PCR using an intron 3/exon 4 primer pair. Immunoprecipitations with SXL or SNF were included as positive controls. Negative controls included precipitations with no antibody, pre-immune serum and Polycomb (PC). The lanes marked input are controls in which the amount of extract corresponds to a percentage of the material used in each Co-IP experiment.

Finally, we asked whether PPS associates with the SXL protein and found that antibodies against the PPS protein can in fact immunoprecipitate SXL (Figure 5A). Interestingly, this interaction was weakened when we carried out these experiments in the presence of RNase. This suggests that the SXL/PPS interaction is mediated and/or stabilized by RNA.

### PPS associates with unspliced *Sxl* RNA

Because the SXL protein exerts its effect by binding directly to its own pre-mRNA, we postulated that PPS might also associate with the unspliced *Sxl* pre-mRNA. To test this idea, we asked whether *Sxl* pre-mRNA is detectable in PPS immunoprecipitates. The results of these RNA immunoprecipitation assays (RIP), which were carried in nuclear extracts without fixation, clearly shows that the unspliced *Sxl* RNA is detectable by RT-PCR using an intron 3-exon 4 primer pair (Figure 5B). In control reactions, we found that *Sxl* RNA was also detected in SXL immunoprecipitates, but not in extracts treated with antibodies against the chromatin binding protein Polycomb (PC) or in pre-immune serum.

To determine whether the SXL protein is required for the association between PPS and the *Sxl* pre-mRNA, we carried out RIP assays in nuclear extracts made from embryos collected from mothers homozygous for a viable allele of *daughterless, da^1^*. *da^1^* mutant mothers produce eggs that lack SXL protein because *SxlPe* is not activated [12]. *SxlPm*, however, is activated, and the resulting transcripts are therefore spliced in the male mode. As illustrated in Figure 5C, PPS was able to co-immunoprecipitate unspliced *Sxl* RNA in these SXL-deficient mutant extracts. In control reactions, we found that *Sxl* RNA was detected in SNF immunoprecipitates, but not in controls. Thus, we conclude that the PPS/*Sxl* pre-mRNA association does not depend on the presence of SXL protein in the extract.

### Recruitment of PPS, SNF, and SXL during transcription

**Figure 6:**
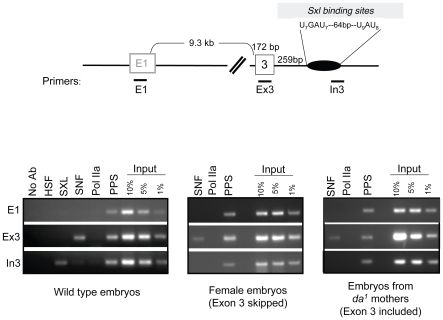
Accumulation of SXL, SNF, and PPS along the body of the *Sxl* gene in embryos. ChIP using SXL, SNF, and PPS specific antibodies. After ChIP, the extracted DNA was analyzed by PCR using primer pairs positioned along the *Sxl* gene as diagramed. Specificity controls include ChIPs using antibodies directed against the Heat Shock Factor (HSF) and RNA Pol IIa (8WG16), as a well as a no antibody control (no Ab). ChIPs were carried out, from left to right, in 8–12 hour old wild-type embryos, female embryos (embryos from *C(1)DX* mothers crossed to *Sxl^7BO^* males) and embryos from *da^1^/da^1^* mothers. To ensure that the PCR reactions of the antibody enriched DNA fell within a linear range of amplification, PCR reactions were carried out on serially diluted input DNA, ranging from 1% to 10% of total DNA. The PCR data shown here are representative of three independent ChIP experiments.

To gain a better understanding of the functional relationship between PPS, SXL and SNF, we compared the dynamics of their recruitment to the nascent *Sxl* transcript by combining genetic analysis with chromatin immunoprecipitation (ChIP) assays (Figure 6). Splicing factor-ChIP assays, which have been used in both yeast and mammalian cells, are possible because many splicing factors are recruited to their RNA targets while still in close contact with template DNA [15]–[17].

To validate this approach, ChIP analysis was first carried out with antibodies against SNF in a sexually mixed population of wild type 8–12 hour embryos. ChIP studies in mammalian cells have shown that U1 snRNP proteins specifically target regions of genes that include 5′ splice sites of recognized exons [17]. This predicts that SNF will accumulate on a region that includes the male-specific third exon (Ex3), but not on the SXL binding site which is located ∼250 bp away in the third intron (In3). As a specificity control, we assayed for SNF accumulation on the first exon of the *SxlPe* transcripts (E1) because in 8–12 hour embryos E1 is treated as an intron, and thus should not be recognized by the splicing machinery. In agreement with our expectations, we found that SNF was present at the third exon (Ex3), but not at the other two locations. Additional controls for specificity include our demonstration that these three regions of the *Sxl* gene were not precipitated in controls or in ChIP assays carried out with the DNA binding Heat Shock Factor (HSF). As a final control for specificity, ChIPs were also carried out with the 8WG16 antibody against the hypophosphorylated form of RNA polymerase II (Pol IIa), because previous studies have shown that Pol IIa does not accumulate within the body of actively transcribed genes [18],[19].

Having shown that recruitment of SNF to the *Sxl* gene can be detected by ChIP, we next asked whether we could use this methodology to view SXL and PPS recruitment. In agreement with *in vitro* RNA binding assays [20], we found that SXL was present at the intronic SXL binding site, In3. PPS, on the other hand, was not only present on the third exon (Ex3) but also localized to the intronic E1 and In3 regions. Together these results argue that PPS, in contrast to both SNF and SXL, is uniformly distributed across the *Sxl* transcription unit.

Next we asked whether the pattern of recruitment is different on nascent transcripts destined to be spliced in the female or male mode. Males do not express SXL protein; therefore, SXL-ChIP of chromatin isolated from a mixed sex population of embryos resulted in the analysis of only female embryos. PPS and SNF, on the other hand, are expressed in both male and female embryos, thus the analysis of chromatin from wild type embryos would mask any sex-specific differences, should they exist. To circumvent this issue, we repeated the SNF and PPS ChIP experiments in two mutant populations of embryos. To exclusively assay *Sxl* transcripts destined to be spliced in the female mode, chromatin was prepared from embryos collected from a stable stock in which all females carry an attached X chromosome and all males carry *Sxl^7BO^*, an X-linked deletion allele of *Sxl*. As there is no *Sxl* DNA present in the male embryos, this analysis is limited to *Sxl* chromatin isolated from female embryos. To generate a population of embryos where all nascent *Sxl* transcripts are destined to be spliced in the male mode, we prepared chromatin from embryos from *da^1^* mothers. As described above, maternal DA protein is required to initiate *SxlPe* transcription early in embryonic development, therefore all eggs laid by homozygous mutant females fail to produce SXL protein. As shown in Figure 6, we found that the pattern of PPS and SNF accumulation was not dependent on the source of the chromatin: PPS accumulated at all three sites, whereas SNF was only detected on the third exon. We therefore conclude that the recruitment pattern of PPS and SNF along the *Sxl* gene is the same in males and females.

### Recruitment of PPS to the *SxlPm* promoter region

**Figure 7:**
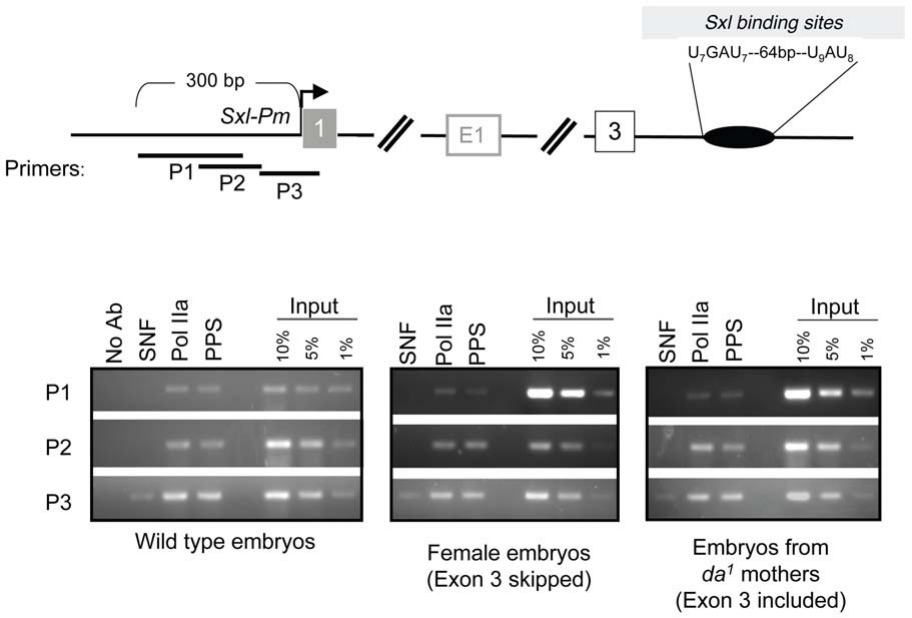
Accumulation of PPS near the *SxlPm* promoter in embryos. ChIP assays using SNF–and PPS–specific antibodies were carried out using the same population of embryos as described in Figure 6. After ChIP the extracted DNA was analyzed by PCR using primer pairs positioned around the *SxlPm* promoter as diagramed. The 8WG16 antibody, which detects the hypophosphorylated Pol II (Pol IIa), is used here to mark the promoter. Consistent with published studies, Pol IIa was largely detected at the promoter whereas SNF was only detected by a primer set designed to detect the beginning of the transcription unit.

The uniform distribution of PPS on the *Sxl* transcription unit, together with its classification in the Gene Ontology Database as a protein involved in transcription, suggested to us that PPS might initially be recruited near *SxlPm*. We therefore repeated the ChIP experiments using two different primer sets targeting sequences upstream of the *SxlPm* transcription start site (P1 and P2) and one that includes the first exon (P3). ChIP studies in *Drosophila* and mammalian cells have shown that the hypophosphorylated form of RNA polymerase II (Pol IIa), detected by the 8WG16 antibody, is highly concentrated at the start of actively transcribed genes [18],[19]. In agreement with these studies, we found that Pol IIa specifically accumulates at P1, P2 and P3 (Figure 7). SNF, as expected, only accumulates on P3, the region that overlaps with the first exon. As shown in Figure 7, we found that PPS accumulates on P1, P2 and P3 and that this distribution is not sex-specific. Taken together, these results suggest that PPS associates with the *Sxl* promoter.

### PPS also targets the SXL–regulated *transformer (tra)* pre–mRNA

**Figure 8:**
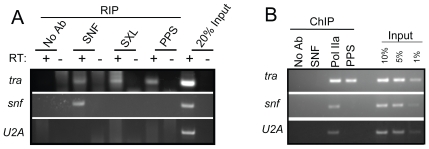
*tra* is a PPS target gene. (A) RIP assays demonstrating that PPS associates with the *tra* pre-mRNA, but not the *snf* pre–mRNA or the intronless *U2A* transcript. The presence of unspliced RNA in the IP pellet was detected by RT–PCR. (B) ChIP assays demonstrating that PPS is detected at the *tra* promoter (identified by Pol IIa accumulation), but not at the *snf* or the *U2A* promoter. The exact position of the primers used in the RIP and ChIP assays are described in the Materials and Methods section.

In addition to its autoregulatory function, the SXL protein also binds the *tra* pre-mRNA to regulate its sex-specific expression [21]. To determine whether PPS is involved in *tra* pre-mRNA splicing, we first carried out RIP assays and found that *tra* pre-mRNA is detectable in PPS immunoprecipitates, as well as in control SXL and SNF immunoprecipitates (Figure 8A). We then carried out ChIP experiments to determine whether PPS is recruited to the *tra* promoter region (Figure 8B). To demonstrate that we had targeted the promoter region, ChIP experiments with antibodies against the hypophosphorylated form of RNA polymerase II (Pol IIa) 8WG16 were used as a positive control. Antibodies against SNF are used here as a negative control. In accordance with our expectations, we found that PPS does in fact associate with the *tra* promoter region.

While these studies clearly suggest that PPS has an additional role in *tra* splicing regulation, it is unlikely that PPS is globally associated with all actively transcribed genes, as we fail to detect associations with the intronless *U2A* gene and the intron containing *snf* gene (Figure 8A and 8B). On the other hand, PPS is clearly not limited to SXL-mediated splicing events because loss of PPS function is lethal to both sexes. What these additional functions are remains to be determined.

## Discussion

**Figure 9:**
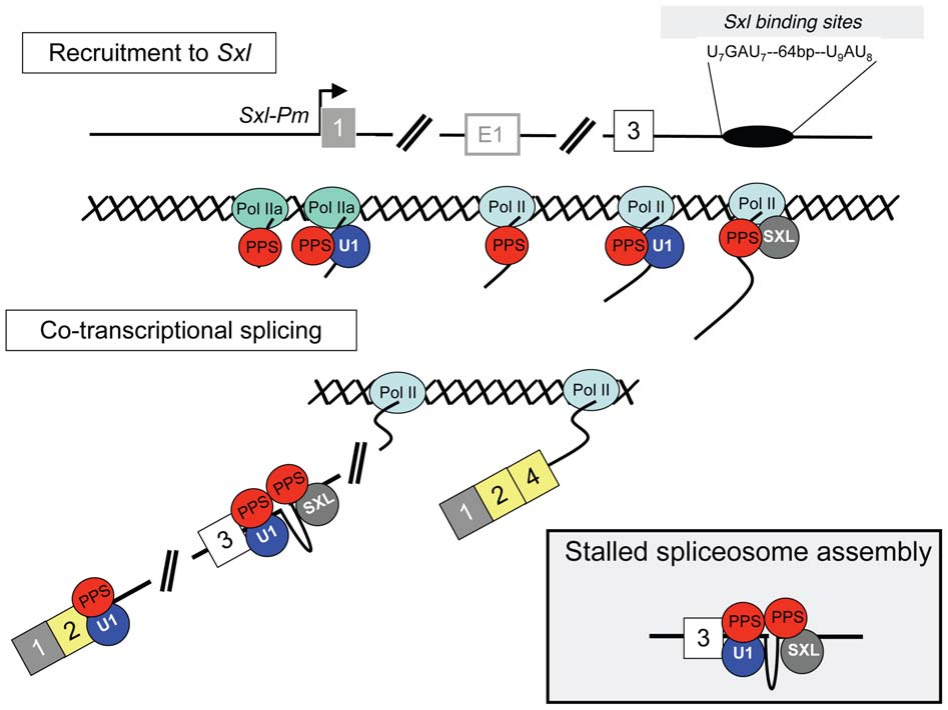
Co-transcriptional model for *Sxl* splicing autoregulation. PPS associates with Pol II during transcription (Pol II, oval) to help recruit the U1 snRNP (U1, blue circle) and SXL (grey circle) to the appropriate locations on the nascent transcript. In addition, PPS may help nucleate the interaction between the U1 snRNP and SXL. Splicing could be blocked immediately (insert) or spliceosome assembly could continue, stalling only later in the pathway. The end result is a dead-end complex that guarantees that the male exon will be skipped, and that exon 2 is spliced to exon 4. In males, where there is no SXL protein, the U1 snRNP is free to assemble into a functional spliceosome and exon 3 is included in the mature transcript (not shown).

Genetic studies have established that SXL protein is both necessary and sufficient to engage the *Sxl* autoregulatory splicing loop [22]. Mechanistically, however, SXL does not act alone and collaborates with components of the general splicing machinery, including the U1 snRNP, to block inclusion of the male exon [2]. In this study, ChIP assays showed that SNF and SXL are specifically recruited to their predicted binding sites on the nascent transcript: SNF to 5′ splice sites and SXL to its intronic binding sites. These data, together with our observation that the recruitment of SNF is not influenced by the presence or absence of SXL, support the current model in which SXL blocks male exon inclusion by interacting with general splicing factors bound to authentic splice sites (Figure 9). Splicing could be blocked immediately, or spliceosome assembly could continue, stalling only later in the pathway. The U1 snRNP, however, is only transiently associated with the spliceosome as it assembles on the splicing substrate and is released before the spliceosome is catalytically active [23]. Therefore it is likely that SXL acts by interrupting spliceosome assembly at some point after splice site recognition by the U1 snRNP, but before catalysis begins.

In studies begun by screening for SNF-interacting proteins, we identified PPS, a conserved and previously uncharacterized *Drosophila* protein, as a novel component of the machinery required for skipping the *Sxl* male exon. We were able to establish this connection by demonstrating that (1) animals carrying loss of function *pps* mutations are compromised in their ability to regulate *Sxl* splicing, (2) PPS associates with the U1 snRNP via a direct interaction with SNF and (3) PPS associates with the SXL protein and the unspliced *Sxl* RNA.

Although physically associated with the U1 snRNP, PPS does not appear to be a general splicing factor because it does not associate with all spliced transcripts (this study), it is not found in affinity-purified *Drosophila* spliceosomal complexes [23] and it is not a homolog of a previously identified human splicing protein [24]. Thus, PPS stands apart from the other proteins known to facilitate proper *Sxl* splicing, all of which are known to be components of the splicing machinery.

The results of our ChIP analysis also distinguishes PPS from known splicing factors, as it reveals a strikingly distinct pattern of accumulation along the *Sxl* gene, including occupancy at the *SxlPm* promoter region. This pattern of accumulation suggests that PPS is loaded onto the RNA at the promoter and/or that it has a role in transcription. Numerous studies have documented physical interactions between the transcriptional machinery and splicing factors [25]. Thus, PPS may well act in concert with the transcription machinery to promote SXL-mediated exon skipping (Figure 9). For example, PPS could serve as a bridging protein to accelerate recruitment of SXL to the nascent transcript, or it could facilitate the formation of the inhibitory SXL/U1 snRNP interaction.

Whether PPS is physically coupled to the transcription machinery and/or has a role in controlling transcription will require additional studies. However, the fact that PPS contains 4 signature motifs typically found in proteins with known functions in transcription adds credence to this idea. Of these 4 motifs, the PHD finger is the most extensively studied. Numerous studies have shown that PHD fingers have histone methylation binding activity. Indeed, PPS is likely to have histone binding activity, as the PHD domains of both the *S. cerevisiae* (BYE1) and mammalian (DIDO) PPS homologs preferentially bind to tri-methylated H3K4 (H3K4me3) *in vitro* [26],[27]. The possibility of a PPS–histone link is further strengthened by the presence of the metazoan specific BRK motif, a domain that is found in only two other *Drosophila* proteins–Brahma and Kismet–both of which are known to be chromatin binding proteins [28],[29]. A connection to transcription is also suggested by the presence of the TFS2M motif. This motif is named after its founding member located in the center of the transcription elongation factor S-II, where it is essential for binding Pol II [30]. Finally, SPOC domains have been identified in a variety of proteins linked to transcription, the best characterized of which is the human SHARP nuclear hormone co-repressor [31],[32]. A conserved function in transcription is particularly compelling in light of the current view that transcription and splicing are mechanistically coupled. In this regard, there are a few well-documented examples of mammalian chromatin binding proteins that affect alternative splicing [33]. For example the H3K4me3 binding protein, CHD1, associates with the spliceosome and is required for efficient splicing [34]. In another example the BRK domain containing chromatin remodeling protein, BRAHMA/BRG1, influences the alternative splicing of several transcripts [35].

Although still speculative, a mechanism linking transcription to splicing regulation is likely to be of major importance in early embryogenesis. Engagement of the autoregulatory splicing loop requires that the initiating source of SXL protein, produced from the transiently expressed *SxlPe* derived transcripts, be present when *SxlPm* is activated so that its transcripts can be alternatively spliced to produce more SXL protein. The changeover from *SxlPe* to *SxlPm* is tightly coordinated and uncoupling these events leads to disruptions in *Sxl* regulation [6],[7]. While these studies suggest that transcriptional regulation of *SxlPm* is important for the switch to autoregulation, our studies lead us to propose that PPS contributes to the success of this switch by concurrently facilitating *SxlPm* transcription and promoting male-exon skipping.

PPS function is not restricted to *Sxl* splicing regulation. In studies designed to test for specificity, we discovered that PPS also associates with the SXL-regulated *tra* pre-mRNA. In addition, we found that *pps* function is essential for viability of both sexes, indicating that *pps* function is not limited to SXL-mediated splicing events and is involved in other developmental pathways. In humans, the PPS homolog DIDO has been linked to a blood disorder called myeloproliferative disease (MPD) [36]. The relevance of this connection is suggested by our finding that homozygous *pps* mutant larvae contain melanotic tumors, tumors that often result from over-proliferation and aggregation of blood cells [37]. Thus, the discovery of PPS' role in controlling alternative splicing may be of significance to additional developmental pathways.

## Materials and Methods

### Yeast two hybrid screen

Using the entire SNF protein as bait, we screened 9.8×10^7^ clones from *Drosophila* embryonic and adult cDNA libraries and identified 78 positive clones, all of which included the C-terminal end of the *pps* (CG6525) gene. PPS was also reported to be a binding partner of CDK7 (CG3319) [38]. However, we have not been able to verify the authenticity of this interaction (data not shown), and suspect that this interaction is based on an annotation error because the *snf* and *cdk7* genes partially overlap [39].

### *Drosophila* genetics

Mutant alleles and deficiencies used in this study include: *Sxl^f1^*, *Sxl^7BO^ da^1^*, *da^2^*, *fl(2)d^2^*, *U2af38^ΔE18^, Df(2Lh)D1* (designated as *spf45^Δ^* in Table 1), *Df(3R)Exel7316*, *PBac{WH}Dip-C^f00706^* and *PBac{WH}CG17202^f01979^* [2]–[4], [8], [12], [40]–[42]. We generated *pps^1^* by FRT-mediated recombination between *PBac{WH}Dip-C^f00706^* and *PBac{WH}CG17202^f01979^* using the conditions described previously [8],[9]. Throughout this analysis we found that the phenotypes of *pps^1^/pps^1^* and *pps^1^*/*Df(3R)Exel7316*, animals to be identical, indicating the absence of confounding background mutations on the *pps^1^* mutant chromosome. The *P{pps+}* and *P{Scg-β+, CG17202^+^}* genomic rescue constructs were generated by standard methods in the pCaSpeR4 transformation vector and transgenic flies were produced at Genetic Services (http://www.geneticservices.com). Functional *P{Scg-β+, CG17202^+^}* transgenes (abbreviated as *P{Scg-β+}* in the text) were selected based on their ability to complement a known point mutation in CG17202. Each transgenic line was then tested for its ability to rescue the different *pps* mutant phenotypes, including the lethality of *pps^1^*/*Df(3R)Exel7316* and *pps^1^/pps^1^* animals. The data presented in this paper are obtained with *P{pps+}* line # 10. Additional marker mutations and balancers used in this study are described on Flybase (http://www.flybase.org).

### Antibodies, GST-pull downs, co-immunoprecipitations, and western Blots

The antibody against PPS was raised in guinea pig by Covance (http://www.covance.com) against a glutathione S-transferase (GST) tagged C-terminal domain PPS fragment (amino acids 1370–2016) purified from bacteria. We note here that this PPS antibody has not proven to be useful for immunohistochemistry. The other antibodies used in this study include mouse anti-SNF-4G3 [43],[44], guinea pig anti-U2A' [45], rabbit anti-U170K-151 [2], mouse anti-SXL-M114 [46], guinea pig anti- HSF [47], rabbit anti-PC [48], and mouse anti-RNA Pol IIa-8WG16 (Millipore, #05-952). Crude extracts for GST-pull down experiments (Figure 1) and Western blots (Figure 4) were prepared from 3–8 hour old embryos, sexed and genotyped third instar larvae or sexed adults in NET buffer (150 mM NaCl, 50 mM Tris, pH 7.5, 5 mM EDTA) supplemented with 0.5% NP-40 and Complete Mini Protease Inhibitor Cocktail Tablets (Roche). Nuclear extracts for co-immunoprecipitation experiments were prepared from 3–18 hour old embryos as described previously [49] using NET buffer supplemented with 0.5% NP-40 for the co-IPs in Figure 4 and 0.05% NP-40 for the co-IPs in Figure 5. For experiments in which the extracts were pretreated with RNase, 1/10 volume of RNase A (10 mg/ml) and 1/20 volume of RNase T1 (100,000 units/ml) were added directly to the extract and incubated overnight at 4°C. Co-immunoprecipitations, Western blot analysis and GST pull down assays were carried out according to standard protocols, using the conditions described previously [2],[4],[50].

### RT–PCR analysis

Total RNA was isolated from ovaries, adults or embryos using TRIzol (Invitrogen) as directed by the manufacturer. To analyze the endogenous *Sxl* splicing products, the first strand synthesis was carried out with 1 µg of RNA, 500 ng/µl random hexamers with the SuperScript II Reverse Transcriptase System (Invitrogen). The PCR reactions, using the High Fidelity Taq system (Roche), were performed in 50 µl volume with 2 µl of the RT reaction with the following primers: GTGGTTATCCCCCATATGGC and GATGGCAGAGAATGGGAC. The PCR conditions were as follows: 94°C for 1 min, followed by 30 cycles of 94°C for 1 min, 55°C for 1 min, and 72°C for 2 min, and a single final step at 72°C extension for 10 min. Products were detected on a 2% agarose gel by staining with ethidium bromide.

### RNA immunoprecipitation (RIP)

RNA/protein complexes were immunoprecipitated from nuclear extracts and diluted to 5 µg/µl in NET buffer (150 mM NaCl, 50 mM Tris, pH 7.5, 5 mM EDTA), supplemented with 0.05% NP-40, Complete Mini Protease Inhibitor Cocktail Tablets (Roche) and RNase inhibitor (100 U/ml) using the conditions described previously [50]. RNA was isolated from the RNA/protein complexes using TRIzol (Invitrogen) as directed by the manufacturer. RNA was resuspended in 20 µl RNase-free water and DNase-treated. cDNA was synthesized with the SuperScript II Reverse Transcriptase System (Invitrogen) using 4 µl of the eluted RNA with a *Sxl* gene specific primer to exon 4 (GATGGCAGAGAATGGGAC; Figure 6) or random hexamers (Figure 8). The PCR reactions, using the High Fidelity Taq system (Roche), were performed in 50 µl volume with 2 µl of the RT reaction with the following primers–*Sxl*: GAGGGTCAGTCTAAGTTATATTCG and GATGGCAGAGAATGGGAC; *snf*: GGGATGTGCGAATGACTAG and GACTGGAGTTGCGTTCAC; *tra*: GATGCCGACAGCAGTGGAAC and GATGGCACTGGATCAGAATCTG; *U2A*: GGTGAAACT AACGCCGGAGC and CTCAGCTCCTGCAGGTTGTTG. PCR conditions were as follows:: 94°C for 1 min, followed by 30 cycles of 94°C for 1 min, 55°C for 1 min, and 72°C for 2 min, and a single final step at 72°C extension for 10 min. 2 µl of the first-round PCR amplification was subjected to a second round of PCR. . Products were detected on a 2% agarose gel by staining with ethidium bromide.

### Chromatin immunoprecipitation (ChIP)

Live embryos were dechorionated with 50% bleach and fixed for 15 min in a 1.8% paraformaldehyde/heptane fixative solution. Chromatin was prepared from 1–2 gram of fixed 8–12 hour old embryos using the conditions described previously [51] and sonicated for a total of 80 seconds (20 sec pulses with a 1 min rest on ice) to produce sheared products of 300 to 400 bp. ChIP assays were performed with a commercially available ChIP assay kit (#17–295; Millipore). Antibodies used for the IP step were diluted 1∶40 (Pol IIa, HSF, PC and PPS) and 1∶20 (SXL and SNF). After purification, the ChIPed DNA samples were resuspended in 30 µl water. Enrichment of specific DNA fragments was analyzed by PCR on 2 µl ChIP material with the following primer sets: For *Sxl*–P1: CGGGGCTCAAAAGACATAAA and GCGTTAGTTAAGACTCAC TCCATTT; P2: CCGTTACGAATCAAGCGAAG and GGCTGGTCACAC TGTTCATT; P3: CAGCCGAGTGCCTAGAAAAA and ACTTTCCTTCTTCGGCAACA; E1: CAAGTCCAACTTGTGTTCAGA and TCGAACAGGGAGTCACAGTAT; Ex3: CGAAAAGCGAAAGACACTC and GTG TCCTCGATTCAAAAACAT; In3: ACATCATGCTTTTCTTAAGTGC and AACGATCCCCCAGTTATATTC. For *U2A*–GGCAGCGAATTG TTTTTCTG and GAATCTTATAGCCGCGCAAA; For *tra*–TGGTCTCCATGGAAAACGAG and TGCAAACACGGTTTCATTTC; For *snf*–AAACACCGGTGCGATAACAT and CGTTTGGTTGGGTAGCATCT. The PCR conditions for *Sxl* primers P1, P2, P3, E1 and Ex3, *tra* and *snf* were as follows: 94°C for 2 min, followed by 25 cycles of 94°C for 30 sec, 53°C for 30 sec, and 72°C for 1 min. The PCR conditions for *Sxl* In3 and *U2A* were as follows: 94°C for 2 min, followed by 25 cycles of 94°C for 30 sec, 55°C for 30 sec, and 72°C for 1 min. Products were detected on a 3% agarose gel by staining with ethidium bromide.
